# Evidence That Intracellular Stages of *Leishmania major* Utilize Amino Sugars as a Major Carbon Source

**DOI:** 10.1371/journal.ppat.1001245

**Published:** 2010-12-23

**Authors:** Thomas Naderer, Joanne Heng, Malcolm J. McConville

**Affiliations:** The Department of Biochemistry and Molecular Biology, University of Melbourne, Bio21 Institute of Molecular Science and Biotechnology, Parkville, Victoria, Australia; Washington University School of Medicine, United States of America

## Abstract

Intracellular parasites, such as *Leishmania spp,* must acquire suitable carbon sources from the host cell in order to replicate. Here we present evidence that intracellular amastigote stages of *Leishmania* exploit amino sugars in the phagolysosome of mammalian macrophages as a source of carbon and energy. *L. major* parasites are capable of using N-acetylglucosamine and glucosamine as primarily carbon sources and contain key enzymes required for conversion of these sugars to fructose-6-phosphate. The last step in this pathway is catalyzed by glucosamine-6-phosphate deaminase (GND), which was targeted to glycosomes via a canonical C-terminal targeting signal when expressed as a GFP fusion protein. Mutant parasites lacking GND were unable to grow in medium containing amino sugars as sole carbohydrate source and rapidly lost viability, concomitant with the hyper-accumulation of hexosamine-phosphates. Expression of native GND, but not a cytosolic form of GND, in *Δgnd* parasites restored hexosamine-dependent growth, indicating that toxicity is due to depletion of glycosomal pools of ATP. Non-lethal increases in hexosamine phosphate levels in both Δ*gnd* and wild type parasites was associated with a defect in promastigote metacyclogenesis, suggesting that hexosamine phosphate levels may influence parasite differentiation. Promastigote and amastigote stages of the *Δgnd* mutant were unable to replicate within macrophages and were either completely cleared or exhibited reduced lesion development in highly susceptible Balb/c mice. Our results suggest that hexosamines are a major class of sugars in the macrophage phagolysosome and that catabolism of scavenged amino sugars is required to sustain essential metabolic pathways and prevent hexosamine toxicity.

## Author Summary

Protozoan parasites belonging to the genus *Leishmania* are transmitted by sandfly vectors and cause a number of important diseases in humans. These parasites proliferate within mature lysosome compartments in macrophages and other phagocytic cells in the mammalian host. How intracellular stages of *Leishmania* survive within this hydrolytic compartment and the extent to which they utilize different carbon sources is poorly defined. Previous studies have suggested that sugar uptake is important for growth, although the nature of these sugars is unknown. In this study we show that *Leishmania* express all of the enzymes needed to degrade the amino sugars, glucosamine and N-acetylglucosamine. We show that a key enzyme in this pathway is sequestered within modified peroxisomes, or glycosomes, and that this localization is essential for growth on amino sugars and avoidance of amino sugar toxicity. This pathway is also required for parasite proliferation within cultured macrophages and for normal infection of highly susceptible mice. Mutant parasites are either completely eradicated or induce small lesions in Balb/c mice after an extended lag period. These findings suggest that amino sugars generated by the lysosomal breakdown of host glycoconjugates are an important carbon source for intracellular stages of *Leishmania*, particularly during the early stages of infection.

## Introduction

A number of microbial pathogens selectively target macrophages and other phagocytic cells during the course of infection of their mammalian hosts [1], [2]. These pathogens can reside within a variety of vacuolar and cytoplasmic compartments from which they must scavenge all of their carbon and nitrogen sources, as well as other essential nutrients [3], [4], [5]. With few exceptions, the biochemical composition of these intracellular niches and the extent to which intracellular pathogens utilize different carbon sources is poorly defined.

*Leishmania* are sandfly-transmitted protozoan parasites that primarily reside in macrophages throughout infection in their mammalian hosts, causing a spectrum of important diseases in more than 12 million people worldwide [6]. Infection of the mammalian host is initiated by flagellated promastigote stages that develop within the mid- and fore-gut of the sandfly vector. Promastigotes injected into the skin during a sandfly bloodmeal are rapidly phagocytosed by neutrophils and macrophages and delivered to the mature phagolysosome where they differentiate to small, non-motile amastigotes [7]. In animal models, the number of infected macrophages increases rapidly during the early stages of infection, eventually plateauing coincident with the formation of loosely structured granulomatous lesions dominated by infected macrophages [8]. In susceptible animals, lesion development and metastasis of infected macrophages to other tissues can lead to death, while in resistant animals a strong proinflammatory (TH1) response leads to lesion cure without sterile immunity [9].

**Figure 1:**
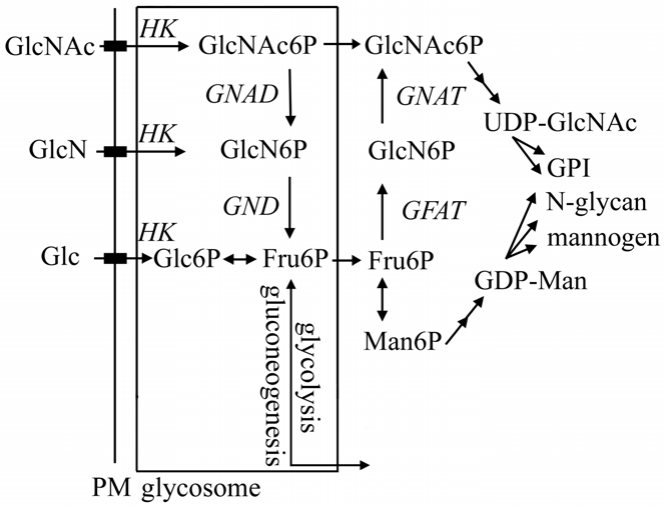
Hexosamine metabolism in *Leishmania*. Exogenous hexosamines (GlcN/GlcNAc) are phosphorylated in the glycosome by a hexose kinase (HK) and either transported to the cytosol for conversion to UDP-GlcNAc or catabolized to Fru6P by the activities of N-acetylglucosamine 6-phosphate deacetylase (NAGD) and glucosamine 6-phosphate deaminase (GND). GND generates fructose-6-phosphate (Fru6P), a key intermediate in carbon metabolism and several pathways of glycoconjugate biosynthesis. The *de novo* synthesis of GlcNAc6P is catalyzed by the cytoplasmic enzymes, glutamine:Fru6P aminotransferase (GFAT) and glucosamine 6-phosphate acetylase (GNAT).

Recent studies have provided insights into the major carbon sources used by intracellular stages of *Leishmania.* A *L. mexicana* mutant lacking three high affinity hexose transporters is unable to establish an infection in macrophages or susceptible mice [10], suggesting that hexose uptake is essential for intracellular growth. However, levels of hexose in the phagolysosome are likely to be limiting for growth as a *L. major* mutant with a defect in gluconeogenesis is also poorly virulent in macrophages and susceptible mice [11]. These studies suggest that intracellular amastigotes depend on both salvage as well as *de novo* synthesis of hexoses from the host niche. The phagolysosome of macrophages could potentially contain a range of different sugars as a result of turnover of host glycans, glycoproteins and proteoglycans [12]. We have recently shown that a *L. major* hexosamine auxotroph is capable of inducing normal lesions in susceptible mice [13], suggesting that the macrophage phagolysosomes contain sufficient levels of the amino sugars, glucosamine (GlcN) or N-acetylglucosamine (GlcNAc), to sustain the minimal hexosamine requirements of intracellular amastigotes. The *Leishmania* genomes also contain a number of genes that are predicted to comprise a functional N-acetylglucosamine (NAG) catabolic pathway raising the possibility that hexosamine sugars may be an important carbon source *in vivo* (Fig. 1). This pathway catalyzes the conversion of GlcNAc to fructose-6 phosphate (Fru6P) via successive uptake, phosphorylation, deacetylation and isomerization-deamination reactions [14] (Fig. 1). A number of pathogenic bacteria (i.e. *Vibrio cholerae*) and fungi (i.e. *Candida albicans*), but not in the non-pathogenic fungi *Saccharomyces cerevisiae* [14], [15] have a functional NAG catabolic pathway (Fig. 1). In *C. albicans*, the NAG catabolic pathway is specifically induced after phagocytosis by macrophages and is important for virulence [16], [17].

In this study we have investigated the role of the putative *L. major* glucosamine-6 phosphate deaminase (GND) in central carbon metabolism and pathogenicity *in vivo*. We show that cultured parasite stages are able to utilize GlcNAc and to a lesser extent GlcN as their major carbon source *in vitro*. We also show that GND is targeted to modified peroxisomes, termed glycosomes and that this localization is essential for growth when hexosamines are the sole carbohydrate source. Finally, we show that GND is important for parasite growth in macrophages and critical for establishment of infection in susceptible animal models. Our findings suggest that intracellular stages of *Leishmania* are dependent on both scavenged hexosamines and *de novo* synthesized sugar phosphates to sustain essential pathways of carbohydrate metabolism.

## Results

### Identification of the NAG-pathway in *Leishmania*

**Figure 2:**
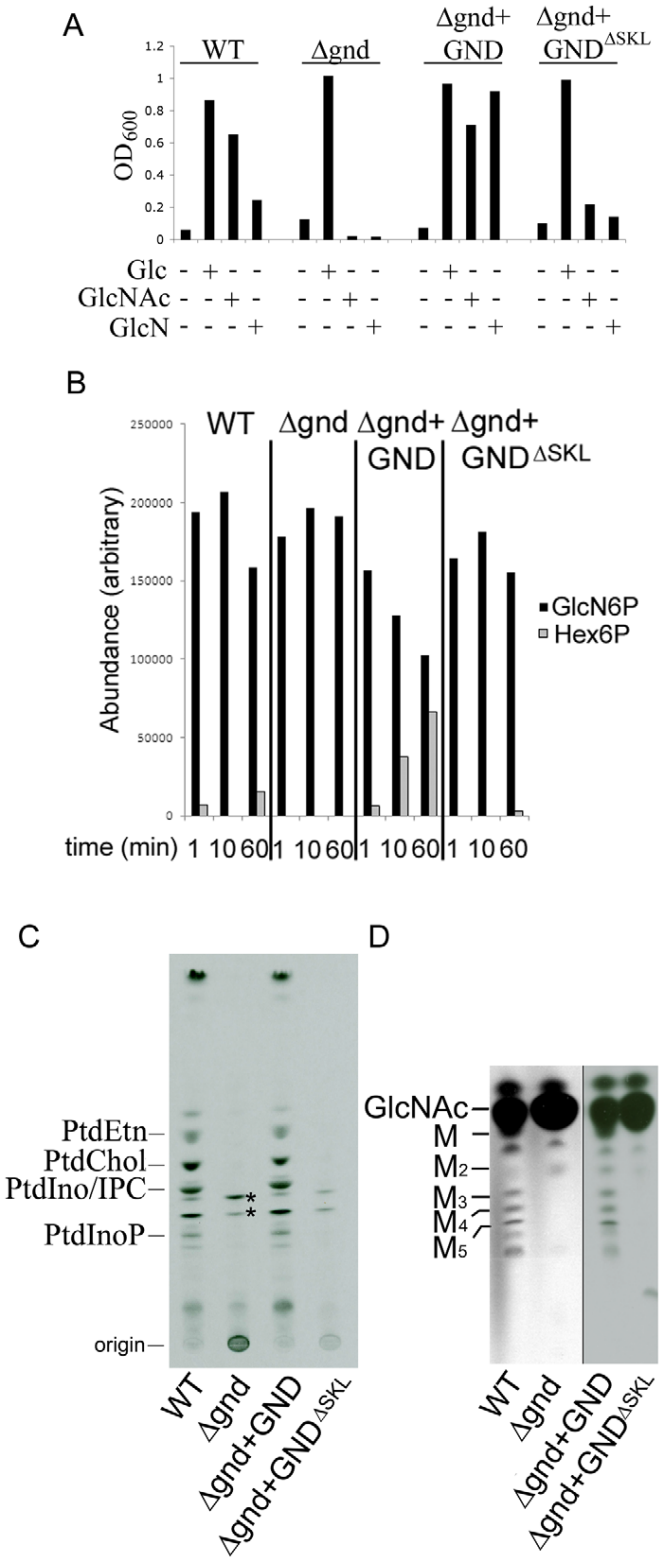
GND is required for growth on GlcN or GlcNAc. (A) Wild type (WT), *Δgnd* and complemented strains (*Δgnd +GND, Δgnd +GND^ΔSKL^* were suspended in CDM with or without Glc, GlcN or GlcNAc (13 mM). The optical density of cultures (OD_600_) at day 4 are shown. (B) *L. major* wild type, Δ*gnd,* Δ*gnd* +GND and Δ*gnd* +GND^ΔSKL^ promastigotes were lysed and GND activity determined by measuring production of hexose-6-phosphates from GlcN6P. (C,D) Wild type, *Δgnd* and complemented strains were labeled with ^3^H-GlcN and incorporation of ^3^H-label into (C) lipids and (D) the carbohydrate reserve polymer mannogen, assessed by HPTLC and fluorography. Major lipid species comprise PtdEtn, PtdCho, PtdIno, inositolphosphoceramide (IPC), PtdInoP and GPI species (asterix); Mannose (M) and mannogen oligomers (M_n_).

We have recently shown that *L. major* can scavenge exogenous GlcN or GlcNAc for the synthesis of essential glycoconjugates [13]. To investigate whether *Leishmania* utilize exogenous hexosamines as a carbon source, *L. major* promastigotes were cultivated in defined medium containing either Glc, GlcN or GlcNAc as sole carbohydrate source. *L. major* promastigotes failed to proliferate in the absence of any sugars although they remain viable for several days (Fig. 2A). Growth was restored when the glucose-free medium was supplemented with Glc, GlcNAc or, to a lesser extent, with GlcN (Fig. 2A), demonstrating that *Leishmania* expresses a functional NAG catabolic pathway and can utilizes hexosamines as the main carbon source. BLAST searches of the *Leishmania* genome (tritrypdb.org) revealed the presence of genes encoding a putative GlcNAc6P deacetylase (NAGD, LmjF36.0040) and a GlcN6P deaminase (LmjF32.3260) indicating the presence of a canonical NAG-catabolic pathway (Fig. 1). These genes shared 22% and 41% identical amino acids to *C. albicans* NagA and GND1, respectively. The genome of the related parasite, *T. cruzi* also contains homologues for NAGD (Tc00.1047053506507.10) and GND (Tc00.1047053511025.50), while *T. brucei* contains a homologue for GND (Tb11.01.8520), but not NAGD (Fig. S1). The absence of NAGD in *T. brucei* is consistent with the recent finding that this parasite can utilize GlcN, but not GlcNAc [13]. None of the trypanosomatid parasites contained identifiable orthologes for hexosamine-specific transporters or kinases, suggesting that the uptake and phosphorylation of hexosamine sugars may be mediated by previously characterized hexose transporters and hexose/gluco-kinase(s) (Fig. 1). Taken together, these data show that *Leishmania* contains a functional NAG catabolic pathway and that GlcNAc/GlcN catabolism is sufficient to sustain parasite growth.

### Characterization of *Leishmania* GND

To further define the role of the NAG catabolic pathway in *Leishmania*, the two chromosomal alleles of the putative *GND* gene in *L. major* were sequentially replaced with nourseothricin and bleomycin resistance cassettes. Several clones were isolated in Glc-rich medium, which contained correct insertion of the resistant cassettes and loss of the *GND* gene as confirmed by PCR analysis (Fig. S2). In contrast to wild type parasites, *Δgnd* promastigotes were unable to grow in medium containing GlcNAc or GlcN as the only source of hexose (Fig. 2A). The Δ*gnd* growth phenotype was completely reversed by ectopic expression of *GND* on the pX-based episome (Fig. 2A). Overexpression of GND in the *Δgnd* mutant markedly improved growth on GlcN (Fig. 2A), indicating that low levels of GND expression in wild type parasites may account for poor growth on GlcN. Consistent with this conclusion, GND enzyme activity in cell extracts of wild type parasite was very low, but was readily detected in the *Δgnd* parasite line overexpressing native GND (Fig. 2B). Loss of GND activity in the *Δgnd* mutant was further confirmed by metabolic labeling with [^3^H]GlcN and analysis of major end-products of central carbon metabolism. Following labeling of wild type parasites with [^3^H]GlcN, label was observed in the major cellular phospholipids and the carbohydrate reserve material mannogen (Fig. 2C, D), reflecting the intracellular conversion of GlcN6P to Fru6P and the redistribution of label into other intermediates in central carbon metabolism. In contrast, none of these metabolites were labeled with [^3^H]GlcN in *Δgnd* parasites (Fig. 2C,D). The residual labeling of GPI lipids in the mutant reflects the direct incorporation of [^3^H]-GlcNAc into these glycolipids (Fig. 2C).

### The glycosomal localization of GND is required for efficient function

**Figure 3:**
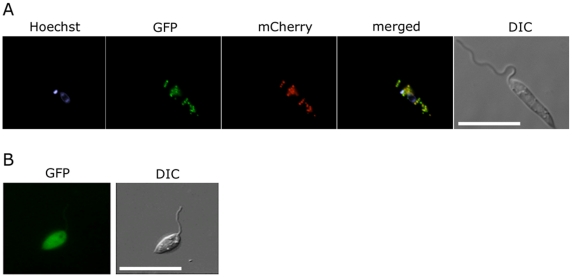
Glycosomal targeting of GND. (A) *L. major* promastigotes were transfected with plasmid encoding GFP-GND and mCherry-FBP (glycosomal marker) protein and the two proteins localized by fluorescence microscopy and Differential interference contrast (DIC) microscopy after labeling with Hoechst dye (nuclear and mtDNA). (B) Localization of the GFP-GND fusion protein lacking the canonical C-terminal tripeptide sequence SKL. Scale bar  = 10 µm.

*L. major GND* is predicted to contain a canonical C-terminal glycosomal targeting signal comprising the tripeptide, SKL. Glycosomal localization was confirmed by expression of a N-terminal GFP chimera of GND in promastigotes. The GFP::GND fusion protein was restricted to punctate structures throughout the parasite cell body co-localizing exactly with the co-expressed glycosomal marker mCherry::FBP fusion protein (Fig. 3A). Significantly, a GFP-fusion protein containing GND without the C-terminal tripeptide SKL (GND^ΔSKL^) exhibited a cytosolic distribution (Fig. 3B) demonstrating that the SKL motif is essential for the glycosomal targeting of GND. To investigate whether glycosomal targeting of GND is required for activity, *Δgnd* parasites were transfected with episomes encoding untagged GND^ΔSKL^. Complementation with GND^ΔSKL^ did not restore normal growth on either GlcN or GlcNAc, although after a lag phase of several days some growth was observed (Fig. 2A). Similarly, metabolic conversion of ^3^H-GlcN into mannogen or lipids was undetectable in the presence of GND^ΔSKL^ (Fig. 2B, C). The glycosomal localization of GND is therefore required for normal growth on hexosamine sugars.

### Growth on hexosamine sugars is cytotoxic for the *L. major Δgnd* mutant

**Figure 4:**
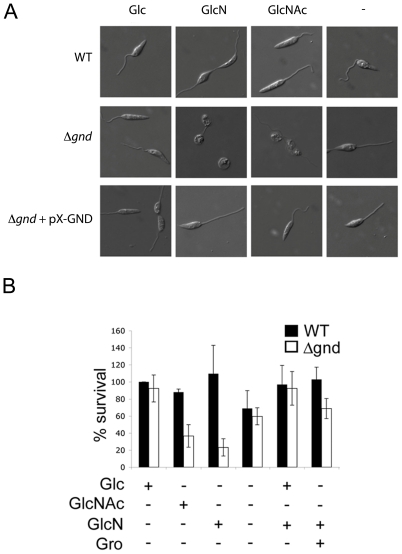
Exogenous hexosamines become toxic in the absence of GND. (A) Wild type, *Δgnd* and *Δgnd* complemented with full length GND or truncated GND (GND^ΔSKL^) were cultivated in M199 medium containing either Glc, GlcN, GlcNAc or no hexoses. Parasite morphology was monitored by DIC microscopy after 24 hr. (B) Wild type and *Δgnd* promastigotes were cultivated in M199 medium with or without indicated sugars for 24 hr. Parasite survival was determined by suspending parasites in complete medium containing glucose and measuring OD_600_ at day 2. Survival is expressed relative to wild type parasites grown in glucose-supplemented media from three independent experiments. Error bar  =  SD. Glycerol is abbreviated as Gro.

**Figure 5:**
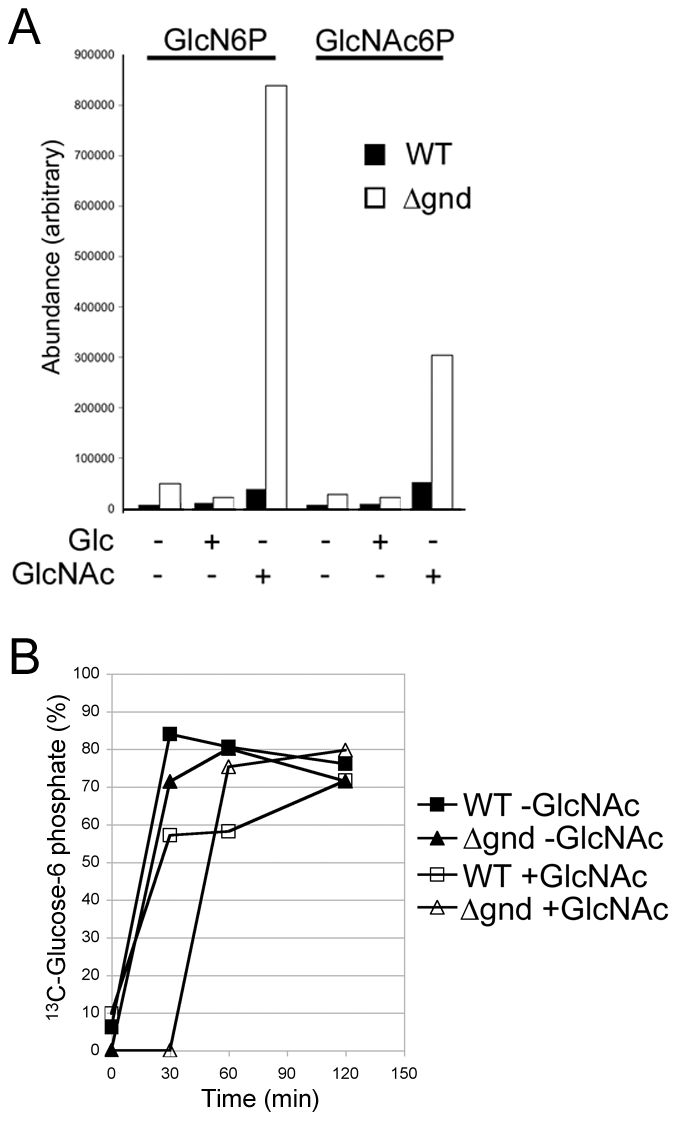
Accumulation of hexosamine phosphates in GlcNAc-fed *Δgnd* parasites. (A) Concentration of intracellular sugar-phosphates in WT and Δ*gnd* parasites after cultivation (4hr) in the presence of Glc or GlcNAc. (B) *L. major* wild type and *Δgnd* promastigotes were suspended in hexose-free CDM supplemented with or without 13 mM GlcNAc. After 4 hr, the medium was supplemented with 13 mM [U-^13^C]-Glc and parasites harvested at the indicated time points. The rate of synthesis of Glc6P (indicated by percent Glc6P labeled with ^13^C), an indicator of glycolytic flux, was determined by GC-MS.

While wild type promastigotes require an exogenous carbohydrate source for growth, they retain a high level of viability when completely starved of hexoses/hexosamines for 24hr (Fig. 4A,B). The *Δgnd* parasites also retain viability when suspended in hexose/hexosamine-free medium, but rapidly lost viability when suspended in medium containing either GlcN or GlcNAc (Fig. 4A,B). Hexosamine toxicity in *Δgnd* parasites was associated with the hyper-accumulation of GlcNAc6P and GlcN6P (Fig. 5A), most likely reflecting the unregulated phosphorylation of internalized sugars by the glycosomal hexose kinase. Hexosamine toxicity was largely abrogated by addition of alternative carbon sources, such as glucose or glycerol (Fig. 4B), that would allow restoration of ATP levels in the glycosome. To further investigate the consequences of elevated levels of GlcNAc6-P on glycosomal metabolism, wild type and *Δgnd* parasites were pretreated with GlcNAc, then metabolically labeled with ^13^C-U-glucose. The *de novo* synthesis of Glc6P (reflecting glycosomal levels of ATP) was then assessed by measuring the incorporation of ^13^C into sugar phosphates by gas chromatography mass spectrometry. Wild type parasites rapidly phosphorylated exogenous glucose whether or not they had been pre-cultivated in medium containing GlcNAc (Fig. 5B). In contrast, *Δgnd* parasites exhibited a marked lag (∼30min) in glucose phosphorylation when pre-incubated in GlcNAc-containing medium, but not when preincubated in Glc/GlcNAc-containing medium (Fig. 5B). These results suggest that hexosamine toxicity arises as a result of the largely unregulated uptake and phosphorylation of exogenous hexosamine sugars by glycosomal hexose kinase, with concomitant depletion of glycosomal pools of ATP. This toxicity is abrogated by the addition of alternative carbon sources that allow net ATP production.

### Promastigote differentiation is modulated by hexosamine-phosphate levels

**Figure 6:**
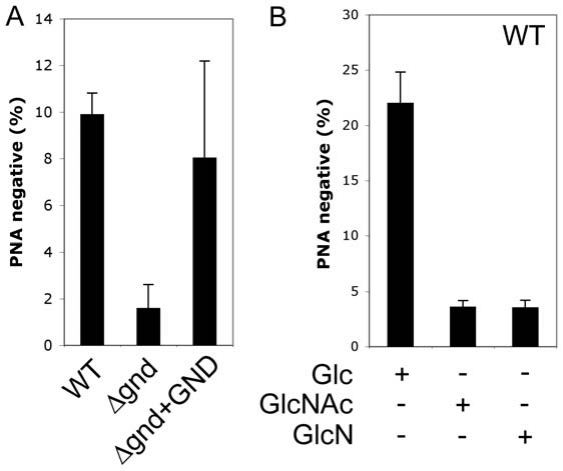
Non-lethal accumulation of hexosamine phosphates leads to a defect in metacyclogenesis. (A) WT, Δ*gnd* and Δ*gnd+*GND promastigotes were cultivated in SDM containing 10% FCS and differentiation to metacyclic stages determined after agglutination with the peanut agglutinin lectin (PNA). Unlike dividing promastigotes, metacyclic promastigotes are not agglutinated by PNA. (B) *L. major* wild type promastigotes were grown in CDM containing either Glc, GlcNAc or GlcN as the sole carbohydrate and metacyclogenesis assessed by PNA agglutination. Error bars represent SED from three independent experiments.

A proportion of *L. major* promastigotes stages differentiate into a highly infectious metacyclic stage as cultures reach stationary phase growth. Metacyclogenesis can be monitored by measuring the extent to which promastigotes are no longer agglutinated with the peanut agglutinin lectin, reflecting structural changes in the surface lipophosphoglycan [18]. While 9.9 ± 0.9 % of wild type promastigotes became PNA-negative in stationary phase, only 1.6 ± 1 % of *Δgnd* promastigotes converted to PNA-negative phenotype (Fig. 6A). Metacyclogenesis was largely restored (8 ± 4%) in the Δ*gnd*+GND cell line (Fig. 6A). Inhibition of metacyclogenesis in the *Δgnd* mutant was associated with a modest increase in intracellular levels of hexosamine-phosphates (5A), presumably reflecting continued *de novo* synthesis from glucose, combined with reduced catabolism. To further investigate this association, wild type promastigotes were cultivated in medium containing either glucose or GlcNAc as sole carbohydrate. As expected, glucose-fed wild type promastigotes containing normal levels of hexosamine phosphate exhibited a high rate of metacyclogenesis (22 ± 2.8 %). In contrast, GlcNAc-fed promastigotes containing elevated levels of hexosamine phosphate (Fig. 5A) displayed a clear defect in metacyclogenesis (3.6 ± 0.6 %) (Fig. 6B). Changes in the intracellular levels of hexosamine-phosphates may thus regulate key differentiation processes in these parasites.

### Attenuated virulence of *Δgnd* parasites in susceptible mice and macrophages

**Figure 7:**
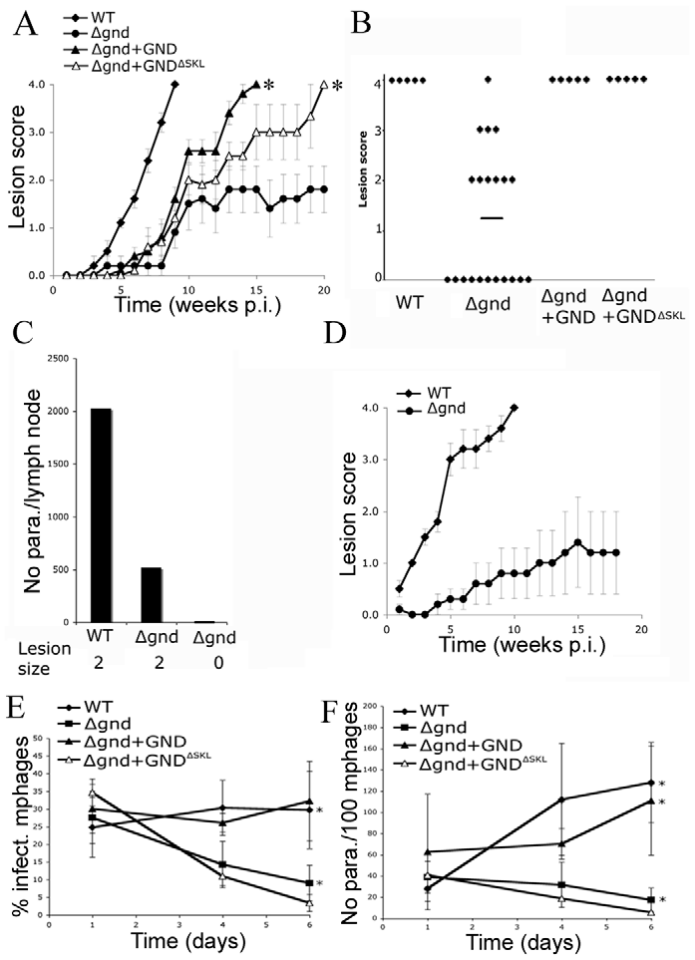
Attenuated virulence of *Δgnd* in mice and macrophages. (A) Wild type, *Δgnd* and complemented *Δgnd* promastigotes were used to infect BALB/c mice intradermally and lesion formation was scored over time (error bar  =  SEM, n = 5). * p<0.01 (Student t-test). (B) Lesion scores in mice infected with wild type, *Δgnd*, and complemented *Δgnd* promastigotes 20 weeks post-infection. The line represents the average of three independent experiments of 15 mice in total. (C) Lesion burden was determined by the limiting dilution assay from draining lymph nodes (parasite numbers are based on 1 million lymph cells). (D) Lesion-derived amastigotes were used to re-infect naïve BALB/c mice and lesions were scored as in (A). RAW 264.7 macrophages were infected with promastigotes of *L. major* wild type, *Δgnd*, *Δgnd +*GND and *Δgnd +*GND^ΔSKL^ and (E) percent infected macrophages and (F) intracellular parasite numbers were determined by microscopy at day 1, 4 and 6 p.i. Error bars represent SED from three independent experiments. * p<0.05 (Student t-test).

To investigate whether hexosamine metabolism is required for *Leishmania* infectivity, BALB/c mice were infected subcutaneously with wild type and *Δgnd* stationary phase promastigotes. Wild type parasites produced large lesions by eight weeks post infection (Fig. 7A,B). In contrast, 50% of the mice infected with *Δgnd* promastigotes failed to develop any lesions (Fig. 7A,B) and viable parasites could not be recovered from the initial inoculation sites or lymph nodes of these mice (Fig. 7C). The rest of the mice developed small lesions over a significantly longer time period (∼8–12 weeks) than wild type parasites (Fig. 7A,B). The parasite burden in the draining lymph nodes of these mice was 4-fold less than in mice infected with wild type parasites (Fig. 7C). To determine whether reduced infectivity was due to selective loss of virulence in the promastigote stage, naïve mice were infected with lesion-derived wild type and *Δgnd* amastigotes. As with promastigote infections, *Δgnd* amastigote infections progressed more slowly than wild type amastigote infections (Fig. 7D). The infectivity of *Δgnd* was largely restored by ectopic expression of GND (Fig. 6A,B). Unexpectedly, the infectivity of the mutant was also partially restored by ectopic expression of truncated GND^ΔSKL^ (Fig. 7A,B). While expression of this construct did not rescue the growth of mutant promastigote stages on hexosamine, it did allow a low level of growth in culture (Fig. 2A). This GND activity appears to be sufficient to at least partially restore normal virulence in the amastigote stage.

**Figure 8:**
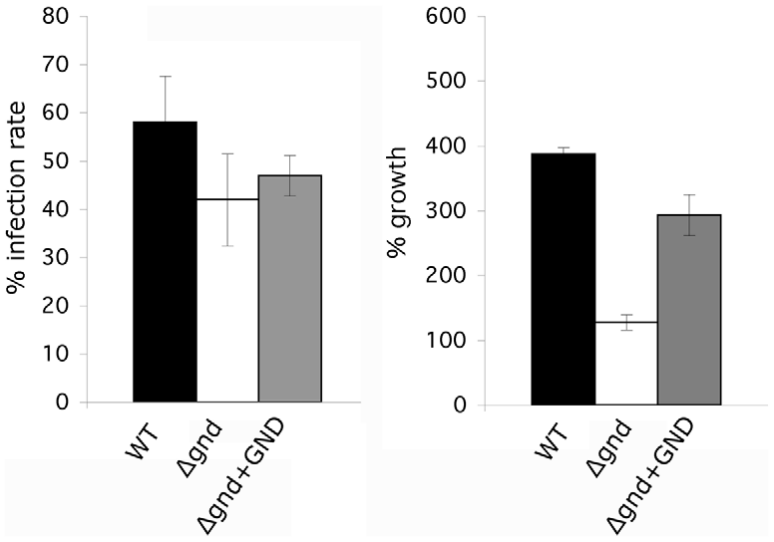
Survival and growth of Δgnd amastigotes in *ex vivo* macrophages. BALB/c mouse peritoneal macrophages were infected with lesion-derived amastigotes of *L. major* wild type, *Δgnd*, complemented *Δgnd* (10∶1 ratio parasites/macrophage). Infected macrophages and intracellular parasite growth (as percentage of number of parasites/100 macrophages compared to day 1) were determined by microscopy at day 4 p.i. (Error bars: SED, n = 3).

To determine whether the attenuated virulence of *Δgnd* parasites in mice was associated with a concomitant decreased capacity to survive and grow in macrophages, RAW 264.7 macrophages were infected with wild type and *Δgnd* stationary promastigotes. Wild type and *Δgnd* parasites were internalized by infected macrophages with equal efficiency (Fig. 7E). However, *Δgnd* parasites failed to grow and were effectively cleared by the macrophages within 6 days, while numbers of wild type parasites increased over the same period (Fig. 7E, F). This phenotype was reversed by ectopic expression of full length GND, but not by cytosolic GND (Fig. 7E, F). Similarly to promastigotes, *Δgnd* lesion-derived amastigotes also failed to replicate in *ex vivo* infected macrophages (Fig. 8). These data suggest that hexosamine catabolism is particularly critical for amastigote survival in naïve, non-activated macrophages both *in vitro* and *in vivo*. Moreover, the fact that lesion-derived *Δgnd* amastigotes are unable to proliferate within cultured macrophages highlights potential differences in hexosamine levels in lesion and cultured macrophages.

## Discussion

Macrophages and other phagocytic cells appear to restrict glucose levels in endocytic compartments, including mature phagolysosomes, in order to prevent growth of microbes [3], [4]. While many bacterial and fungal pathogens are able to survive in macrophages by exploiting alternative carbon sources, such as lipids and amino acids [19], *Leishmania* parasites appear unable to proliferate in the absence of a hexose source despite having an active gluconeogenic pathway [11], [20]. In this study we show that catabolism of the amino sugars via the NAG pathway is important for growth in macrophages and establishment of infection in the mammalian host.

Our study has provided the first detailed analysis of hexosamine catabolism in *Leishmania*. These parasites lack identifiable homologues of other eukaryotic hexosamine sugar transporters or kinases suggesting that GlcN and GlcNAc are internalized by the previously characterized hexose transporters and phosphorylated by glycosomal hexose/gluco-kinases [13]. Glycosomal pools of GlcNAc6P are subsequently catabolized by the combined action of a putative NAGD and the GND characterized in this study (Fig. 1). *Leishmania* lacking *GND* were unable to grow in medium containing hexosamines as the sole source of sugar or to catabolize exogenous [^3^H]-glucosamine, indicating that *GND* encodes the only GlcN6P deaminase activity in these parasites. Interestingly, much higher concentrations of GlcN than GlcNAc are required to sustain maximal growth rates, despite the fact that both sugars are internalized at similar rates [13]. It is possible that GlcNAc is more efficiently phosphorylated by hexose kinase than GlcN [13], or that GlcNAc and GlcN differentially regulate the activity of GND [21], [22], resulting in different rates of utilization.

*Leishmania* GND was localized to the peroxisome-like glycosomes that contain all of the enzymes involved in the upper part of glycolysis and the final steps in gluconeogenesis [11], [23], [24], [25]. The glycosomal localization of GND was critical for parasite growth on GlcN/GlcNAc and for preventing hexosamine toxicity. Hexosamine toxicity was associated with the hyper-accumulation of hexosamine-phosphates, cell swelling and eventual lysis. The accumulation of hexosamine phosphates reflects the absence of normal allosteric feedback mechanisms in the glycosomally located hexose kinases [23], [24], [25] and likely leads to the depletion of glycosomal ATP levels and/or osmotic disruption of this organelle. In support of this notion, hexosamine toxicity was prevented by addition of alternative carbon sources, such as glucose and glycerol that are catabolized in the glycosome with net production of ATP. Direct evidence that hexosamine-phosphate accumulation resulted in selective depletion of glycosomal ATP levels was provided by the finding that phosphorylation of ^13^C-glucose in *Δgnd,* but not wild type parasites, was delayed following growth in hexosamine-containing medium. Significantly, expression of a cytosolic form of GND in the *Δgnd* mutant did not prevent hexosamine toxicity. While our previous studies suggest that hexosamine-phosphates can equilibrate across the glycosome membrane [13], this flux appears to be insufficient to prevent the build-up of hexosamine sugars in glycosomes. In addition to preventing hexosamine toxicity, the glycosomal localization of GND may also be required to avoid the futile cycling of hexosamine and hexose-phosphates in the cytosol. Specifically, *Leishmania* express a cytosolically located glutamine:fructose-6-phosphate amidotransferase that is required for the *de novo* synthesis of GlcN6P from Fru6P (Fig. 1). Futile cycling of *de novo* synthesized GlcN6P to Fru6P by cytosolic GND could lead to the depletion of cellular pools of the sugar nucleotide UDP-GlcNAc and reduced synthesis of essential glycoconjugates [13].

Deletion of *gnd* resulted in an unusual attenuated virulence phenotype in the BALB/c model. In approximately half the infections, mutant parasites were completely cleared, while in the remaining infections, small lesions were induced after a delay of several weeks. This phenotype was reproducible, regardless of whether mutant promastigote or lesion-derived amastigotes were used, indicating that the delayed lesion phenotype was not due to the loss of stage-specific factors or to the evolution of suppressor strains. The poor growth of *Δgnd* parasites in *ex vivo* infected macrophages and attenuated virulence in the BALB/c animal strongly suggests that intracellular amastigotes are dependent on the uptake and catabolism of hexosamine sugars for normal growth in the phagolysosome compartment. While previous studies have suggested that both promastigote and amastigote stages need to take up exogenous sugars for normal growth *in vitro* and *in vivo* [26], the nature of these sugars has been poorly defined. The uptake and catabolism of amino and neutral sugars is likely to be required to sustain fluxes through essential pathways such as N-glycosylation [13], the pentose phosphate pathway [27], inositol synthesis [28], and catabolism of the major carbohydrate reserve material, mannogen [29], [30]. Interestingly, amastigotes also need to synthesize sugar *de novo* via the gluconeogenic pathway [11]. The fact that gluconeogenesis is required for both the establishment of infection and induction of lesions suggests that sugar levels in the phagolysosome are generally low at all stages of infection. In the light of these observations it is curious that surviving *Δgnd* parasites are able to eventually produce small lesions. It is possible that hexosamine sugars constitute the major sugar type in neutrophils and non-activated macrophages encountered during the early stages of infection, while other sugars may become more abundant in macrophages at later stages of infection, providing a more permissive nutrient environment for *Δgnd* parasites. Alternatively, amastigote requirements for hexose/hexosamine may be higher during early stages of infection, but decrease at later stages, reflecting differences in growth rate [8]. The latter possibility was supported by the finding that complementation of the *Δgnd* mutant with the cytosolic GND^ΔSKL^ construct led to a significant restoration of virulence in BALB/c mice. While expression of this construct in *Δgnd* promastigote stages was unable to restore normal growth on amino sugars *in vitro*, a low rate of growth was observed that may be sufficient to sustain the energy needs of lesion amastigotes. Similarly, we have shown that *L. major* amastigote GlcN auxotrophs need significantly less amino sugars for viability than promastigotes [13].

While hexosamine/hexose starvation is likely to be a major contributor to the *Δgnd* loss of virulence phenotype, other factors could contribute to reduced parasite viability *in vivo*. For example, hexosamine toxicity arising from the hyper-accumulation of GlcN(Ac)6P could occur if the phagolysosomes of infected host cells lacked other carbon sources needed to restore the glycosome energy balance. Even modest increases in the intracellular levels of GlcN6P and GlcNAc6P could alter the physiological state of parasites and virulence *in vivo*. Intriguingly, sub-lethal increases in hexosamine-phosphates in both *Δgnd* and wild type parasites inhibited the differentiation of PNA-positive promastigotes to PNA-negative metacyclic promastigotes in stationary phase cultures. Metacyclic promastigotes are preadapted for life in the mammalian host and defects in metacyclogenesis could contribute to loss of virulence of *Δgnd* promastigote stages. However, lesion-derived *Δgnd* amastigotes exhibited a similar attenuated virulence phenotype in BALB/c mice, suggesting that other factors are equally or more important. It remains unclear how changes in intracellular pools of hexosamine phosphates could impact on parasite differentiation. Alterations in the cellular levels of hexosamine-phosphates and UDP-GlcNAc have been shown to regulate mammalian growth and differentiation, either by altering the extent to which many cytosolic and nuclear proteins are modified with O-GlcNAc, or by altering the repertoire of surface expressed N-glycans [31], [32]. In the pathogenic fungi, *Candida albicans*, defects in GlcNAc uptake or catabolism also impact on differentiation (hyphae formation) and virulence [16], [17]. However, neither *Candida albicans* or *Leishmania* modify cytosolic proteins with O-GlcNAc or synthesize complex N-glycans [13], [33]. It is possible that the intracellular levels of these metabolites are directly sensed by signaling pathways involved in regulating parasite growth and differentiation. In this respect, it is notable that elevated levels of tetrahydrobiopterin also inhibit metacyclogenesis in *L. major* [34].

These and previous findings [13] support the conclusion that hexosamines are amongst the most abundant sugars in the *Leishmania* occupied phagolysosome. Hexosamines are major components in a number of host glycoconjugates that are continuously delivered to macrophage lysosomes. For example, macrophages constitutively internalize a range of extracellular proteoglycans and glycoaminoglycans that are rich in GlcN(Ac) [12], [35]. Other GlcN(Ac)-containing host molecules could be delivered to the parasite vacuole via autophagy or glycan turnover pathways [12], [36]. These glycoconjuates are sequentially degraded by a range of host lysosomal endo and exoglycosidases [35]. *Leishmania* spp also secrete a chitinase that could be involved in facilitating the degradation of GlcN/GlcNAc-containing glycoconjugates delivered to amastigote-occupied lysosomes [37]. Such as function is supported by the previously unexplained finding that overexpression of the *L. mexicana* chitinase enhances lesion development in mice [37], [38].

Other trypanosomatid parasites, including the important human pathogen *T. cruzi*, are predicted to express a NAG catabolic pathway. Mammalian-infective stages of *T. cruzi* reside within the lysosome and cytoplasm of macrophages and other host cells [39] and may scavenge non-phosphorylated and/or phosphorylated amino sugars in these niches. In contrast, *T. brucei* contains a putative GND but lacks a putative GlcN6P de-N-acetylase. The absence of a full NAG catabolic pathway in this parasite correlates with a limited capacity to internalize amino sugars [40]. Only GlcN is taken up to any extent and with very low affinity (K_m_ ∼14 mM) [41]. The acquisition of a full NAG catabolic pathway in *Leishmania* and *T. cruzi* may thus have preceded a relaxation in the substrate specificity of their hexose transporters, allowing these parasites to colonize new niches within their respective insect and mammalian hosts.

## Materials and Methods

### Ethics statement

Use of mice was approved by the Institutional Animal Care and Use Committee of the University of Melbourne (ethics number 0811011.1). All animal experiments were performed in accordance with the Australian National Health Medical Research council (Australian code of practice for the care and use of animals for scientific purposes, 7^th^ Edition, 2004, ISBN: 1864962658).

### Parasite strains and cell culture

Promastigotes of *L. major* (MHOM/SU/73/5ASKH) were cultured at 27°C in semi-defined medium-79 (SDM) supplemented with 10% FCS (Gibco, Invitrogen) or in completely defined media (CDM) [11]. For isolation of transfected parasites, the media was supplemented with bleomycin (5 µg/ml; Calbiochem) and/or nourseothricin (70 µg/ml; Werner Inc.) and colonies isolated from SDM-agar (1% Noble Agar, Nunc.) plates.

### Generation of untagged and GFP-tagged GND and mCherry-tagged FBP

The *L. major GND* gene was amplified from *L. major* wild type genomic DNA with the primers *gnd*F (GCCCCCGGGATGCGG ATCGTGATCTCC) and *gnd*R (GGGGATCCCTACAGCTTCGAATAGGCAC) and cloned into pXG1a, and *gfp-gnd*F (GGCGGCCGCATGCGGATCGTGATCTCC) and *gnd*R for cloning into pXG-GFP+2/. Truncated *GND* constructs lacking the SKL sequence (GND^ΔSKL^) were generated by amplification of the *L. major GND* gene using the primers *gnd*F and *gnd^Δskl^*R (GGGGATCCCTAATAGGCACGATTCATGT) and cloned as described above. The constructs were verified by diagnostic digests and DNA sequencing. The restriction sites are underlined. For expression of mCherry-tagged FBP, mCherry was PCR amplified from pRSET-mCherry (a generous gift from Roger Tsien) with primers MJM963 (GCGCCCGGGATGGTGAGCAAGGGCGAG) and MJM967 (TTGAGATCTGCTTGTACAGCTC) and cloned into vector pXG-GFP-FBP using SamI and BglII sites. The mCh::FBP chimera was subsequently liberated with SmaI and BamHI and cloned into pXG-SAT.

### Analysis of glycolipids and mannogen

Mid log phase promastigotes were washed once in PBS and resuspended in glucose-free RPMI medium (2×10^8^ cell/ml). D-[6-^3^H]-GlcN (50 µCi/ml 38.3 Ci/mmol; Perkin Elmer) was added and cells incubated for 30 min at 27°C. Parasites were harvested by centrifugation and glycolipids extracted in chloroform:methanol:water (1∶2∶0.8 v/v) [42]. Labeled glyclipids were analysed by high-performance thin-layer chromatography (HPTLC) using Silica Gel 60 aluminium-backed HPTLC sheets (Merck) developed in chloroform:methanol:13 M ammonium:1 M acetic acid:water (180∶140∶9∶9∶23 v/v) and detected with autoradiography after coating with EnHance spray (New England Nuclear) [43]. Mannogen oligomers were extracted and detected by high-pH anion exchange chromatography as previously described [29].

### *In vitro* activity of GND

*L. major* wild type, Δ*gnd,* Δ*gnd* +GND and Δ*gnd* +GND^ΔSKL^ promastigotes were suspended in hypotonic buffer (1 mM NaHEPES, pH 7.4, 2 mM EGTA, 2 mM DTT, 40 µl/ml PIC) and chilled on ice for 10 min before being lysed by sonication (2×4 sec). Following lysis, samples were centrifuged (25,000 rpm, 0°C, 10 min) to separate organellar and cytosolic fractions. Pellet fractions (3×10^7^ cell equivalents) were suspended in 90 µl assay buffer (50 mM NaHEPES pH 7.4, 2 mM EGTA, 5 mM MgCl_2_, 0.1 % Triton-X 100, 1 mM DTT, 40 µl/ml PIC), containing 1 mM GlcN6P and incubated at 27°C for 1, 10, 30 or 60 min. The reaction was stopped by addition of chloroform:methanol (1∶2 v/v) and extracts dried under nitrogen before phase partitioning in 1-butanol:water (2∶1 v/v). Polar metabolites in the lower aqueous phase were analysed by liquid chromatography-mass spectrometry using a Agilent QTOF instrument.

### Measurment of glycosomal hexose phosphorylation

*L. major* wild type and Δ*gnd* promastigotes were suspended in CDM with or without 13 mM GlcNAc as sole carbohydrate source and cultivated for 4 hr at 27°C. The medium was supplemented with 13 mM [U-^13^C]-Glc and parasites harvested at indicated time points. Parasite metabolism was quenched at each time point by immersing the culture flask (one per time point) in an ethanol-dry ice bath resulting in rapid chilling of the culture suspension to 0°C (∼10 sec) without freezing. Aliquots of the chilled parasite suspension (4×10^7^ promastigotes) were removed and centrifuged in a microfuge (12,000× *g*, 20 sec, 0°C) and the cell pellet washed three times with phosphate buffered saline (0°C) prior to extraction in chloroform:methanol:water (1∶3∶1 v/v) [42]. Water was added to the extract to give a ratio of chloroform:methanol:water (1∶3∶2 v/v), and polar metabolites in the upper aqueous phase derivitized by methoximation and trimethylsilylation [42]. Levels of ^13^C/^12^C- Glc6P at each time point were determined by gas chromatography-mass spectrometry as previously described [42].

### Fluorescence microscopy

Live *L. major* promastigotes expressing GFP::GND chimeras and mCherry::FBP were harvested by centrifugation (800× *g* for 10 min at 25°C), resuspended in PBS containing 8 µg/ml Hoechst (Molecular Probes) for 5 min and overlaid onto poly-L-lysine-coated coverslips. Images were acquired by using a Zeiss Axioplan2 imaging microscope, equipped with Axicam MRm camera and the AXIOVISION 4.3 software (Zeiss) and the montage generated in Photoshop Elements v6 (Adobe).

### Macrophage and mouse infections

Infection of RAW 264.7 macrophages with stationary phase, non-opsinized promastigotes (ratio of 10 parasites/macrophage) or lesion-derived amastigotes (ratio one parasite/macrophage) were performed as described recently [13]. Female BALB/c mice (6–8 weeks old) derived from a pathogen-free facility (Bio21 Institute, University of Melbourne). Intradermal injections and isolation of parasites were performed as described else where [13]. Lesions were analyzed and scored as previously described [44] and the parasite burden was determined from total lymph node using the limiting dilution assay [13]. The draining lymph node was harvested and lymph cells were liberated through a mesh sieve. 1×10^5^ cells from each cell suspension were suspended in SDM-79 medium containing 10% FCS and titrated in a 96-well plate, using threefold dilutions. After 7 days in culture at 27°C, the highest dilution containing parasites was determined and the parasite burden per 10^6^ lymph cells was calculated.

## Supporting Information

Figure S1 Sequence alignment of GND. GND protein sequences of *L. major* (UniProt: Q4Q4U6), *T. cruzi* (Q4D0F2), *T. brucei* (D0AAS0), *E. coli* (B7LKT5), *Y. pestis* (A4TNY0), *H. sapiens* (P46926) and *C. albicans* (Q04802) were aligned with ClustalW and edited with Boxshade, whereby identical or similar residues are boxed in black or grey, respectively. The arrow marks the residue His143 in *E. coli*, involved in catalysis. Residues marked with * are part of the allosteric site of GND as determined by studies performed in *E. coli* [45].
(1.80 MB TIF)

Figure S2 Gene deletion of GND in *L. major* promastigotes. (A) Strategy for targeted gene replacement via homologous recombination. The knock out cassettes for targeted gene deletion of the *L. major GND* was generated by PCR amplifying the 5′UTR of *GND* using the primers *gnd*5′F (GGAAGCTTCTGCGCGTATGCCTCTGCAC) and *gnd*5′R (GGCGAATTCGGTCGATAAAAGTATGTGAA) and by amplification of the 3′ untranslated region (3′UTR) of *GND* using the primers *gnd*3′F (GGGGATCCGTTGCGCCCGCGTGCAAGCA) and *gnd*3′R (GGGGATCCCTACAGCTTCGAATAGGCAC). The *Hind*III/*Eco*RI-digested 5′UTR was cloned into the *Hind*III and *Eco*RI sites of pBluescript II SK (Stratagene), before cloning the 3′UTR into the *Bam*HI and *Xba*I sites. The bleomycin and noureseothricin resistant cassettes were obtained from pXG-BLEO and pXG-SAT, respectively, by digesting with XhoI, followed by blunt-end treatment using Klenow polymerase (New England BioLabs), heat inactivation and digestion with *Bam*HI. The resistant cassettes were isolated by gel purification and cloned between the 5′ and 3′UTR using the SmaI and BamHI sites of the pBluescript vector. The complete knockout constructs were verified by diagnostic digests and DNA sequencing. The *BLE-* and *SAT*-containing *GND* gene replacement cassettes were excised from the plasmid by *Hind*III/*Xba*I digestion, gel-purified and 5 µg transfected into *L. major* promastigotes as described previously [13]. (B) PCR strategy to determine correct integration of knockout constructs. I-VIII denotes primers designed either outside the cloning region or specific for the resistance cassettes. (C) PCR analysis of *L. major* wild type and Δ*gnd* null mutant genomic DNA to check for integration of resistance cassettes and loss of GND gene. Primers used are as follows; lane 1: I, VII; lane 2: I, VIII; lane 3: V, VI; lane 4: IV, V; lane 5:II, III.
(1.09 MB TIF)
