# Supplementary figures and images for: Evidence That Intracellular Stages of Leishmania major Utilize Amino Sugars as a Major Carbon Source
Source: PLoS Pathog. 2010 Dec 23;6(12):e1001245. doi: 10.1371/journal.ppat.1001245 (PMC3009595; doi:10.1371/journal.ppat.1001245)

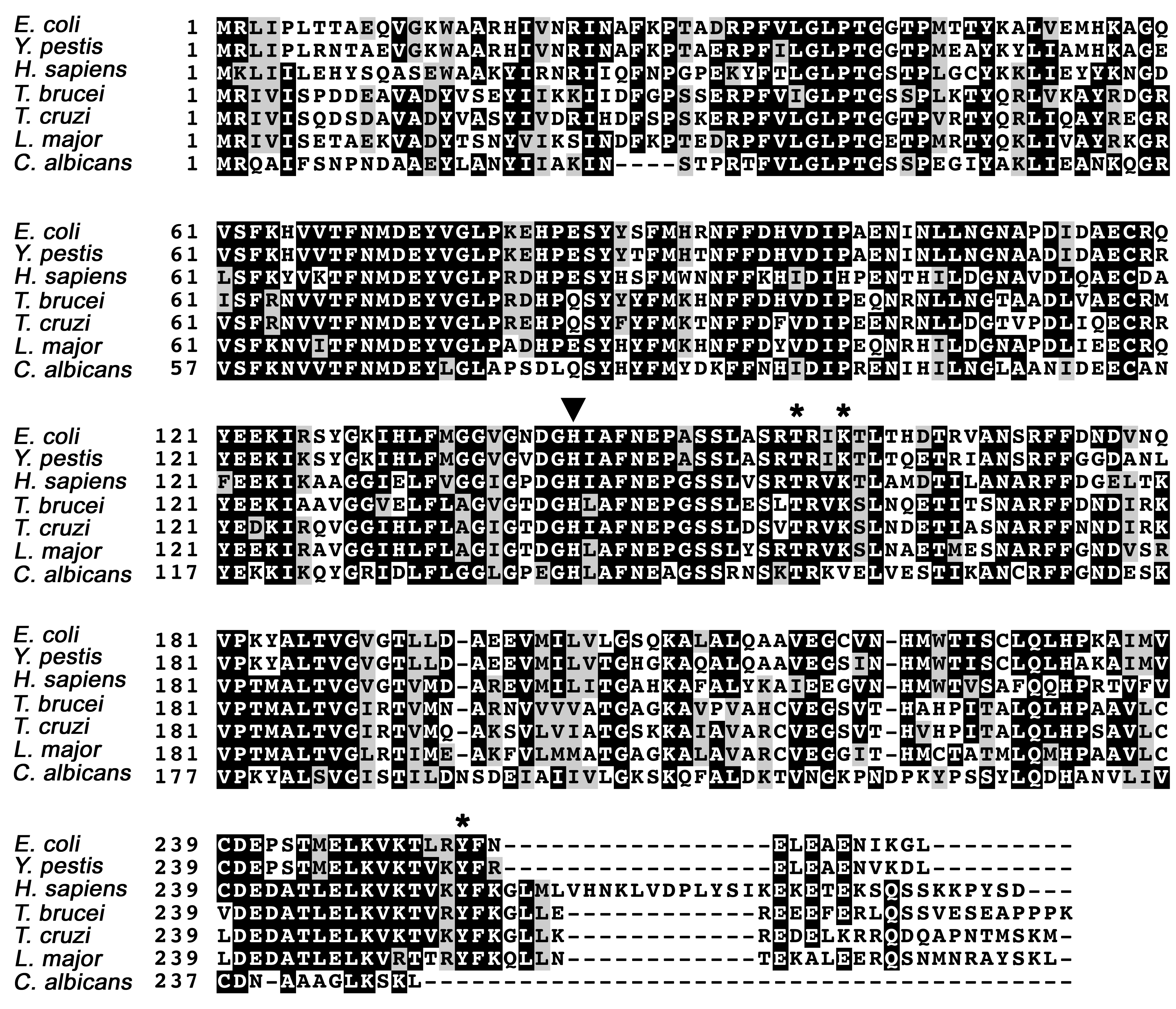

Supplement: Figure S1 — Sequence alignment of GND. GND protein sequences of L. major (UniProt: Q4Q4U6), T. cruzi (Q4D0F2), T. brucei (D0AAS0), E. coli (B7LKT5), Y. pestis (A4TNY0), H. sapiens (P46926) and C. albicans (Q04802) were aligned with ClustalW and edited with Boxshade, whereby identical or similar residues are boxed in black or grey, respectively. The arrow marks the residue His143 in E. coli, involved in catalysis. Residues marked with * are part of the allosteric site of GND as determined by studies performed in E. coli [45]. (1.80 MB TIF) [file ppat.1001245.s001.tif]

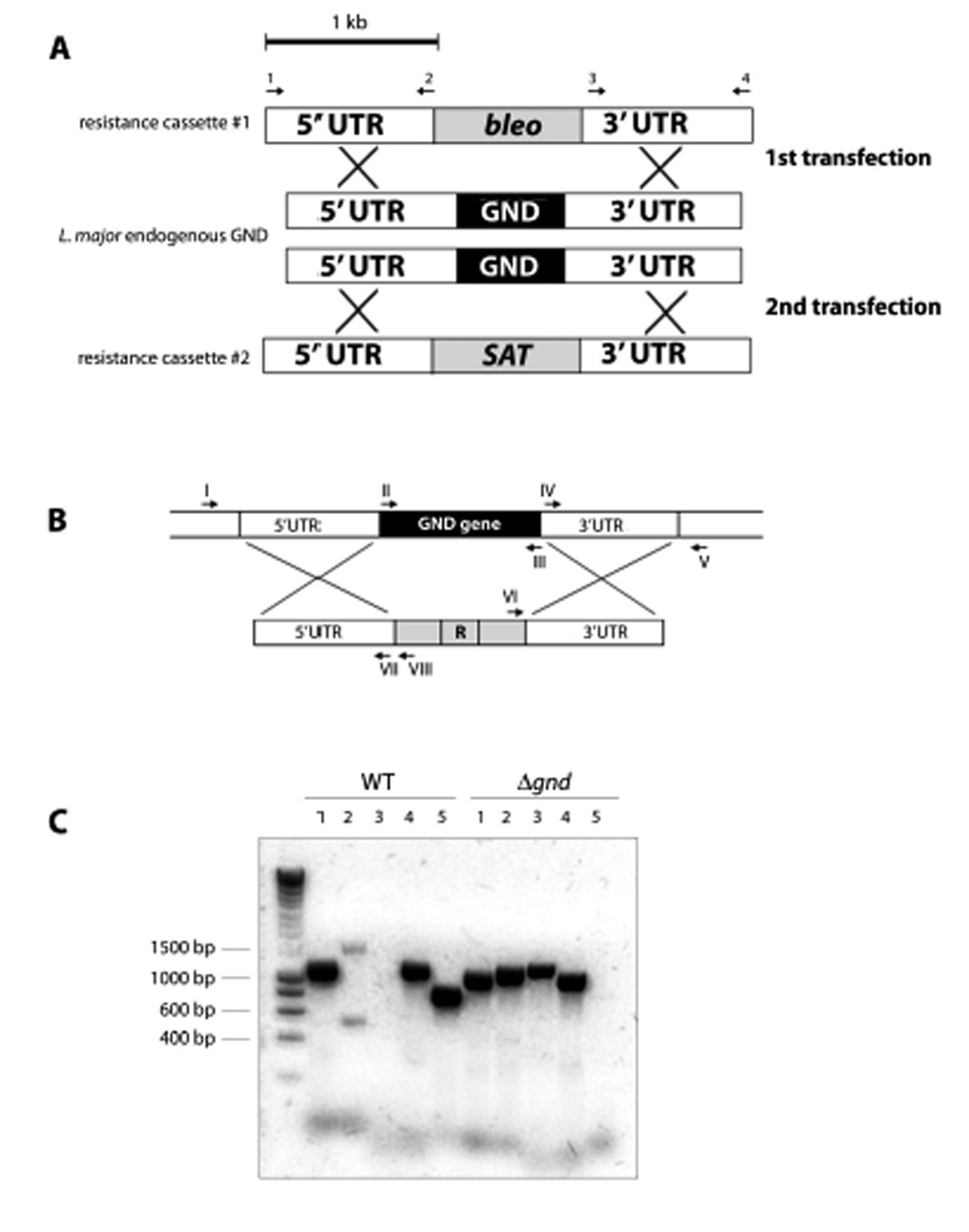

Supplement: Figure S2 — Gene deletion of GND in L. major promastigotes. (A) Strategy for targeted gene replacement via homologous recombination. The knock out cassettes for targeted gene deletion of the L. major GND was generated by PCR amplifying the 5′UTR of GND using the primers gnd5′F (GGAAGCTTCTGCGCGTATGCCTCTGCAC) and gnd5′R (GGCGAATTCGGTCGATAAAAGTATGTGAA) and by amplification of the 3′ untranslated region (3′UTR) of GND using the primers gnd3′F (GGGGATCCGTTGCGCCCGCGTGCAAGCA) and gnd3′R (GGGGATCCCTACAGCTTCGAATAGGCAC). The HindIII/EcoRI-digested 5′UTR was cloned into the HindIII and EcoRI sites of pBluescript II SK (Stratagene), before cloning the 3′UTR into the BamHI and XbaI sites. The bleomycin and noureseothricin resistant cassettes were obtained from pXG-BLEO and pXG-SAT, respectively, by digesting with XhoI, followed by blunt-end treatment using Klenow polymerase (New England BioLabs), heat inactivation and digestion with BamHI. The resistant cassettes were isolated by gel purification and cloned between the 5′ and 3′UTR using the SmaI and BamHI sites of the pBluescript vector. The complete knockout constructs were verified by diagnostic digests and DNA sequencing. The BLE- and SAT-containing GND gene replacement cassettes were excised from the plasmid by HindIII/XbaI digestion, gel-purified and 5 µg transfected into L. major promastigotes as described previously [13]. (B) PCR strategy to determine correct integration of knockout constructs. I-VIII denotes primers designed either outside the cloning region or specific for the resistance cassettes. (C) PCR analysis of L. major wild type and Δgnd null mutant genomic DNA to check for integration of resistance cassettes and loss of GND gene. Primers used are as follows; lane 1: I, VII; lane 2: I, VIII; lane 3: V, VI; lane 4: IV, V; lane 5:II, III. (1.09 MB TIF) [file ppat.1001245.s002.tif]
